# Genetic Characterization of *smg-8* Mutants Reveals No Role in *C. elegans* Nonsense Mediated Decay

**DOI:** 10.1371/journal.pone.0049490

**Published:** 2012-11-16

**Authors:** Jacqueline Rosains, Susan E. Mango

**Affiliations:** Department of Molecular and Cellular Biology, Harvard University, Cambridge, Massachussetts, United States of America; German Cancer Research Center, Germany

## Abstract

The nonsense mediated decay (NMD) pathway degrades mRNAs bearing premature translation termination codons. In mammals, SMG-8 has been implicated in the NMD pathway, in part by its association with SMG-1 kinase. Here we use four independent assays to show that *C. elegans smg-8* is not required to degrade nonsense-containing mRNAs. We examine the genetic requirement for *smg-8* to destabilize the endogenous, natural NMD targets produced by alternative splicing of *rpl-7a* and *rpl-12*. We test *smg-8* for degradation of the endogenous, NMD target generated by *unc-54(r293),* which lacks a normal polyadenylation site. We probe the effect of *smg-8* on the exogenous NMD target produced by *myo-3::GFP*, which carries a long 3′ untranslated region that destabilizes mRNAs. None of these known NMD targets is influenced by *smg-8* mutations. In addition, *smg-8* animals lack classical Smg mutant phenotypes such as a reduced brood size or abnormal vulva. We conclude that *smg-8* is unlikely to encode a component critical for NMD.

## Introduction

The nonsense mediated decay (NMD) pathway is an evolutionarily conserved mRNA surveillance mechanism that recognizes and degrades transcripts bearing premature translation termination codons [1], [2]. In *C. elegans*, mRNAs that have acquired a nonsense mutation or an extended 3′ untranslated region (UTR) are targeted for NMD [3]. In addition, physiological transcripts that carry an early stop codon are substrates for NMD [4], [5], [6]. The central players of the NMD pathway were discovered in *S. cerevisiae* [7] and *C. elegans* [1], [3], [8], [9] and comprise SMG-2/Upf1, SMG-3/Upf2 and SMG-4/Upf3. In worms, *Drosophila* and mammals, the NMD core components are modulated by additional SMG factors. Recent screens have also identified and confirmed new candidate NMD proteins in *C. elegans, H. sapiens* and *D. rerio* [10], [11], [12].

Elegant studies in several organisms have suggested a model in which NMD reflects a competition between SMG-2/UPF1 and Poly(A) Binding Protein (PABP) for ribosome-associated translation release factors. In mammals, the locations of mRNA splice junctions are marked by exon-junction complexes (EJC) during mRNA processing [1]. If the ribosome encounters a premature termination codon (PTC) during the pioneer round of translation, the SURF complex, (consisting of SMG-1, UPF1 and the release factors eRF1 and eRF3) interacts with the EJC, triggers UPF1 phosphorylation by SMG-1 and initiates NMD [13]. In *C. elegans*, EJC components are dispensable for NMD, and PTCs are instead distinguished from normal stop codons by the size of the 3′UTR [10], [14]. Once the PTC has been recognized, target mRNAs are destroyed by either the SMG-6 endonuclease (mammals, *Drosophila* and probably worms) [15], [16], and/or a SMG-5/SMG-7-dependent exonuclease (mammals, yeast and probably worms) [17], [18].

Recently, Yamashita and colleagues used immunoprecipation of HeLa cell lysates to identify proteins that interact with SMG-1, a phosphatidylinositol kinase-related protein kinase [19], [20]. SMG-1 bound two novel, conserved proteins, FLJ23205 and FJL12886, which were renamed SMG-8 and SMG-9 [21]. This pair bound strongly to each other, and modified SMG-1 kinase activity *in vitro*. Inactivation of SMG-8 and SMG-9 lead to a partial stabilization of ß-globin mRNAs in mammalian cell culture, suggesting they might play a role in NMD. The authors also used RNA interference (RNAi) to inactivate *C. elegans smg-8* and *smg-9*, and concluded that *smg-8,* but not *smg-9,* contributed to NMD in worms [21].

**Figure 1:**
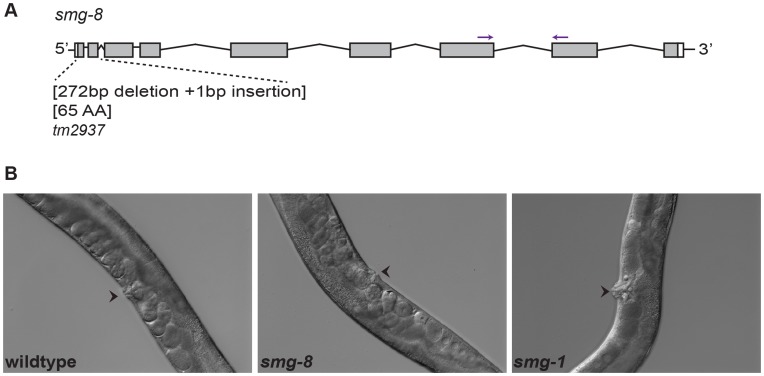
*smg-8* lacks the vulva phenotype associated with mutations in other NMD genes. (A) Schematic representation of the *tm2937* allele, which contains a 272 bp deletion and a 1 bp insertion. This deletion encompasses 22 bp upstream of the start site and the first two exons. Arrows indicate primers used for RT-qPCR (B) Vulval protrusion is one of the phenotypes of canonical *smg* genes. Left panel shows a wildtype vulva, middle panel shows a *smg-8(tm2937)* mutant and right panel shows a *smg-1(r861)* mutant. *smg-8* mutants are similar to wild-type and not to *smg-1*. Arrowheads denote the vulva.

Here we examine *C. elegans smg-8* using a newly generated mutant allele. *smg-8(tm2937)* contains a 272 bp deletion and a 1 bp insertion within *smg-8* (Figure 1A). This deletion encompasses 22 bp upstream of the start site, the initiator ATG and the first two exons. Using animals homozygous for this allele, we employed four approaches to investigate a possible role for *smg-8* in the NMD pathway. Our findings suggest that *smg-8* is unlikely to be a key component for NMD in *C. elegans.*

## Results and Discussion

### *smg-8* Mutants do not Exhibit Phenotypes Associated with NMD Mutants

For our first assay, we examined two of the classical phenotypes associated with *smg* genes. Hodgkin and colleagues reported a reduced brood size of 174 (range 147–211) for *smg-1,* which was lower than the mean brood size of wild-type animals (327, range 270–373) [9]. We observed a mean brood size of 301 (range 242–367, n = 10) for *smg-8(2937),* similar to the mean brood size of our wild-type strain (279, range 211–328, n = 10) (Table 1). In addition, animals bearing a mutation in a canonical *smg* gene have a protruding vulva due to morphological defects [9]. However, vulvae appeared normal in *smg-8(tm2937)* worms (Figure 1B). These data suggest that *smg-8(tm2937)* animals lack two overt phenotypes associated with canonical *smg* mutants.

**Table 1:** Brood Size Comparison of *smg-1* and *smg-8 vs* wild-type.

Strain	Mean Brood Size	Range
*smg-1(r861)* *	174	174–211
Wildtype*	327	270–373
*smg-8(tm2937)*	301	242–367
Wildtype	279	211–328

Average progeny (n = 10 mothers) at 20°C. Progeny were counted every day until no more progeny were observed.

* Data from [9].

### *smg-8* does not Show an NMD Phenotype for the Native NMD Target *rpl-7a* and *rpl-12*

**Figure 2:**
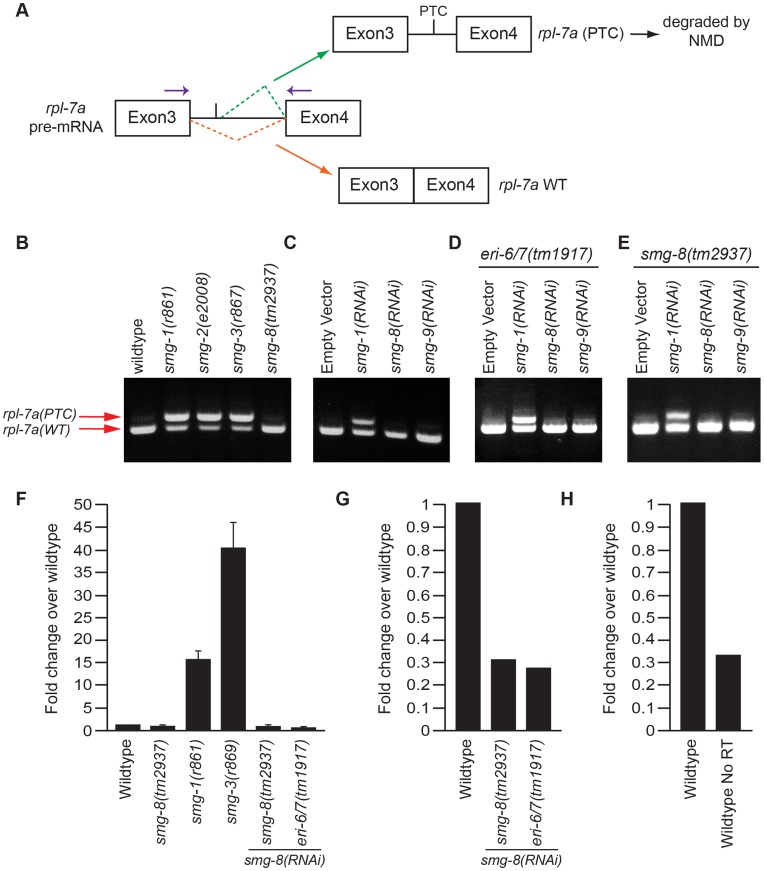
*smg-8* lacks an NMD phenotype for the native NMD target *rpl-7a*. (A) Schematic representation of the two alternatively spliced isoforms of *rpl-7a.* The isoform containing the premature termination codon (PTC) is subject to degradation by NMD, whereas the shorter isoform is not. RT-PCR was performed using a pair of primers that distinguish the two spliced isoforms (purple arrows). (B) The upper, PTC band is visible only when the NMD pathway is compromised by *smg-1, smg-2* or sm*g-3* mutations (lanes 2, 3 and 4). Only the lower WT band is observed in wild-type (lane 1) and *smg-8* mutant (lane 5) animals. (C) Wild-type worms were fed bacteria expressing dsRNA targeting *smg-1*, *smg-8* or *smg-9* from the Ahringer dsRNA library [26]. RNA was analyzed as in (B). (D) An enhanced RNAi mutant strain *eri-6/7* [22], [23] was used and RNAi conducted as in (C). RNA was analyzed as in (B). (E) As in D, using the *smg-8(tm2937)* mutant strain. (F) RT-qPCR using primers flanking the PTC-containing isoform of *rpl-7a,* mRNA levels were calculated using the delta-delta-CT method, relative to the control gene *pmp-3* [27]. Fold enrichment of the PTC mRNA was normalized to 1 for wild-type. The *smg-1* and *smg-3* mutants show an enrichment of 15 and 38 fold, respectively. In contrast, in *smg-8* mutants, the accumulation of the PTC containing isoform is similar to wild-type (0.7 fold enrichment). *smg-8* and *eri-6/7* mutant worms treated with *smg-8* RNAi show 0.7 and 0.4 fold enrichment, respectively. (G) RT-qPCR to quantify *smg-8* RNA. mRNA levels were calculated using the delta-delta-CT method, relative to the control gene *pmp-3* [27]. Fold enrichment was normalized to 1 for wild-type. *smg-8* and *eri-6/7* worms treated with *smg-8* RNAi show 0.3 and 0.26 fold enrichment, respectively. (H) As in (G) for wild-type animals and a negative control that lacked Reverse Transcriptase (No RT). Fold enrichment was normalized to 1 for wild-type. No RT control shows 0.3 fold enrichment.

**Figure 3:**
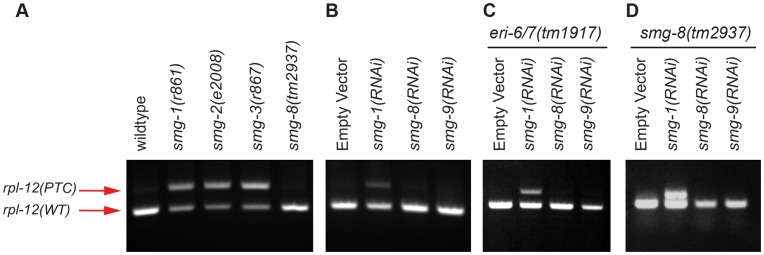
*smg-8* lacks an NMD phenotype for the native NMD target *rpl-12*. (A) RT-PCR was performed using a pair of primers that distinguish the two spliced isoforms of *rpl-12*; the upper, PTC band is visible only when the NMD pathway is compromised by *smg-1, smg-2* or sm*g-3* mutations (lanes 2, 3 and 4). Only the lower, WT band is observed in wild-type (lane 1) and *smg-8* mutant (lane 5) animals. (B) Wild-type worms were fed bacteria expressing dsRNA targeting *smg-1*, *smg-8* or *smg-9* from the Ahringer dsRNA library [26]. RNA was analyzed as in (A). (C) As in (B), using an enhanced RNAi mutant *eri-6/7* [22], [23]. (D) As in (B), using the *smg-8(tm2937)* mutant strain.

For our second assay, we examined transcripts for two ribosomal proteins *rpl-7a* and *rpl-12,* which are natural NMD targets [4]. These genes each generate two alternatively spliced mRNAs, one of which contains a premature termination codon (PTC; Figure 2A). When NMD is active, the longer isoform containing the PTC is degraded and only the shorter isoform accumulates. When the NMD pathway is compromised, the isoform containing the PTC is stabilized, and both mRNA isoforms accumulate (Figure 2A). Using RT-PCR primers that flank the PTC, it is possible to distinguish between the two transcripts [4]. *smg-8* shows no NMD phenotype by this assay. When a known component of the NMD pathway, such as *smg-1*, *smg-2* or *smg-3*, is mutated, the mRNA isoform containing the PTC is stabilized, generating a robust upper band (Figure 2B). In contrast, for *smg-8(tm2937)*, a very faint upper band was observed, comparable to that of the wild-type strain (Figure 2B). To extend this result, we inactivated *smg-8* and also *smg-9* using RNAi, which reduced *smg* mRNA levels at least five-fold (Figure 2G) and similar to a No Reverse Transcriptase negative control (Figure 2H). The results were again negative for NMD (Figure 2C). RNAi is not always robust; therefore we repeated the assay using the strain *eri-6/7(tm1917),* which enhances RNAi [22], [23], and once more observed no NMD phenotype (Figure 2D). Finally, to exclude the possibility that the *tm2937* allele was hypomorphic, we treated *smg-8(tm2937)* mutants with *smg-8* RNAi or *smg-9* RNAi, but again we observed no NMD phenotype (Figure 2E). Similar results were observed for *rpl-12* (Figure 3).

To quantify these data, we used RT-qPCR to measure the increase of the PTC containing isoform. We observed a fold enrichment of 15 and 38 in *smg-1* and *smg-3* mutants respectively, compared to the wild-type (Figure 2F). The PTC containing isoform in *smg-8* mutants remained similar to the wild-type (0.7 fold enrichment). A virtually identical result was obtained with *smg-8* RNAi treatment of *smg-8* or *eri-6/7* mutant worms (0.7 and 0.4 fold enrichment respectively) (Figure 2F). Together, these data reveal that inactivation of *smg-8* fails to stabilize two natural NMD targets, *rpl-7a* and *rpl-12*.

### *smg-8* is not Required for Endogenous NMD in *C. elegans*

**Figure 4:**
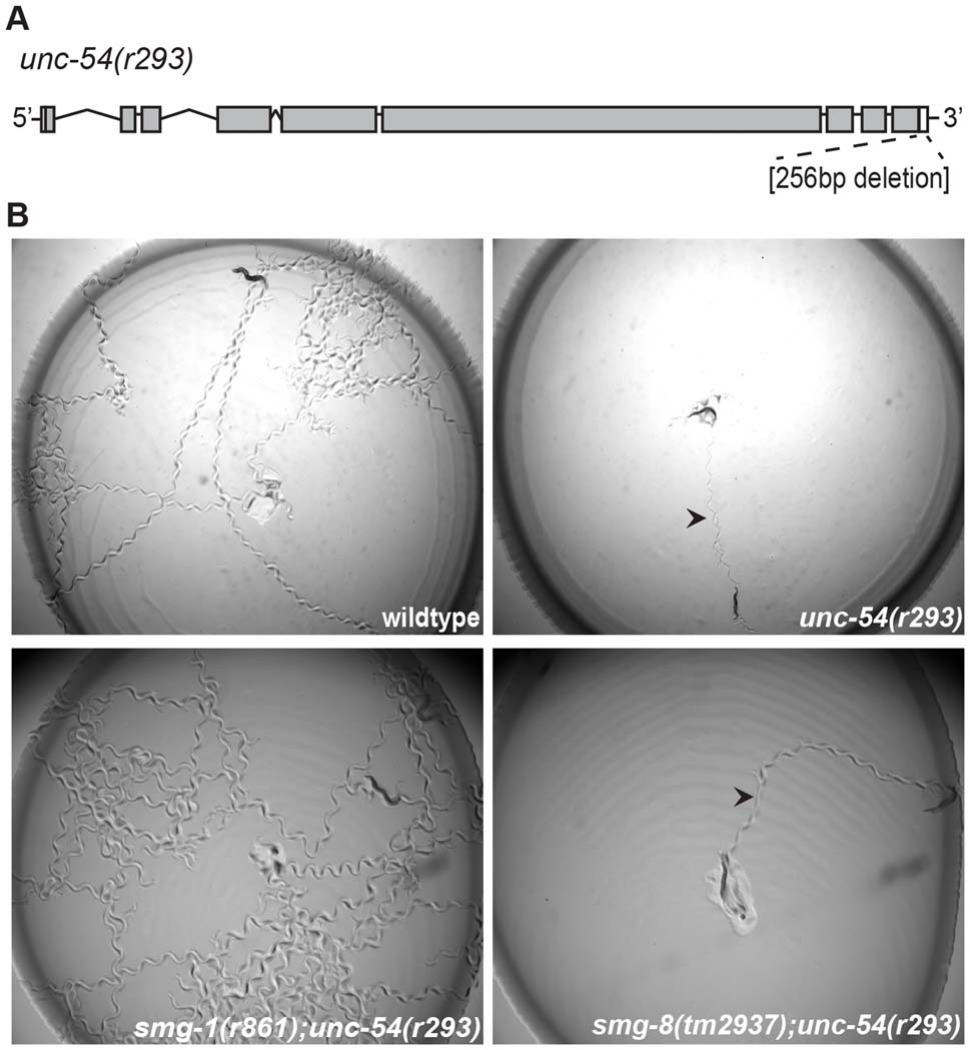
*smg-8* is not required for endogenous NMD in *C. elegans.* (A) *unc-54* gene schematic. The *r293* allele contains a 256 bp deletion within *unc-54* that includes the 3′ cleavage and polyadenylation site. (B) *smg-8(tm2937)* or *smg-1(r861)* mutations were combined with *unc-54(r293).* Two worms were placed in the middle of the bacterial lawn and allowed to crawl for 45 minutes. Wild-type worms that crawl (top left) leave tracks in the lawn whereas *unc-54(r293)* mutants cannot move well (top right). *smg-1(r861)* suppresses *unc-54(r293)* mRNA degradation and restores movement [3] (bottom left). In contrast, *smg-8* does not suppress the paralysis phenotype (bottom right). Arrowheads indicate the tracks left by paralyzed worms.

As a third test for NMD, we examined an endogenous target of the NMD pathway: *unc-54(r293)* [3], [9]. The *unc-54* gene generates a muscle myosin heavy chain (MHC) in *C. elegans*, and the *unc-54(r293)* allele contains a 256 bp deletion that removes the normal 3′ cleavage/polyadenylation site and most of the 3′UTR [3] (Figure 4A). This deletion causes the production of a long *unc-54* mRNA transcript that terminates at a cryptic poly(A) site and renders *unc-54* an NMD target. Without the MHC, *unc-54* mutant worms are paralyzed (Figure 4B). In the absence of NMD components such as *smg-1*, the *unc-54(r293*) mRNA is stabilized, wild-type protein is produced and the Unc phenotype is suppressed [3] (Figure 4B). We generated *unc-54(r293)*; *smg-8(tm2937)* double mutants and observed no suppression of the paralysis phenotype (Figure 4B), consistent with our hypothesis that *smg-8* is not required for NMD in *C. elegans*.

### *smg-8* does not Restore Expression the Exogenous NMD Target *myo-3*::GFP

**Figure 5:**
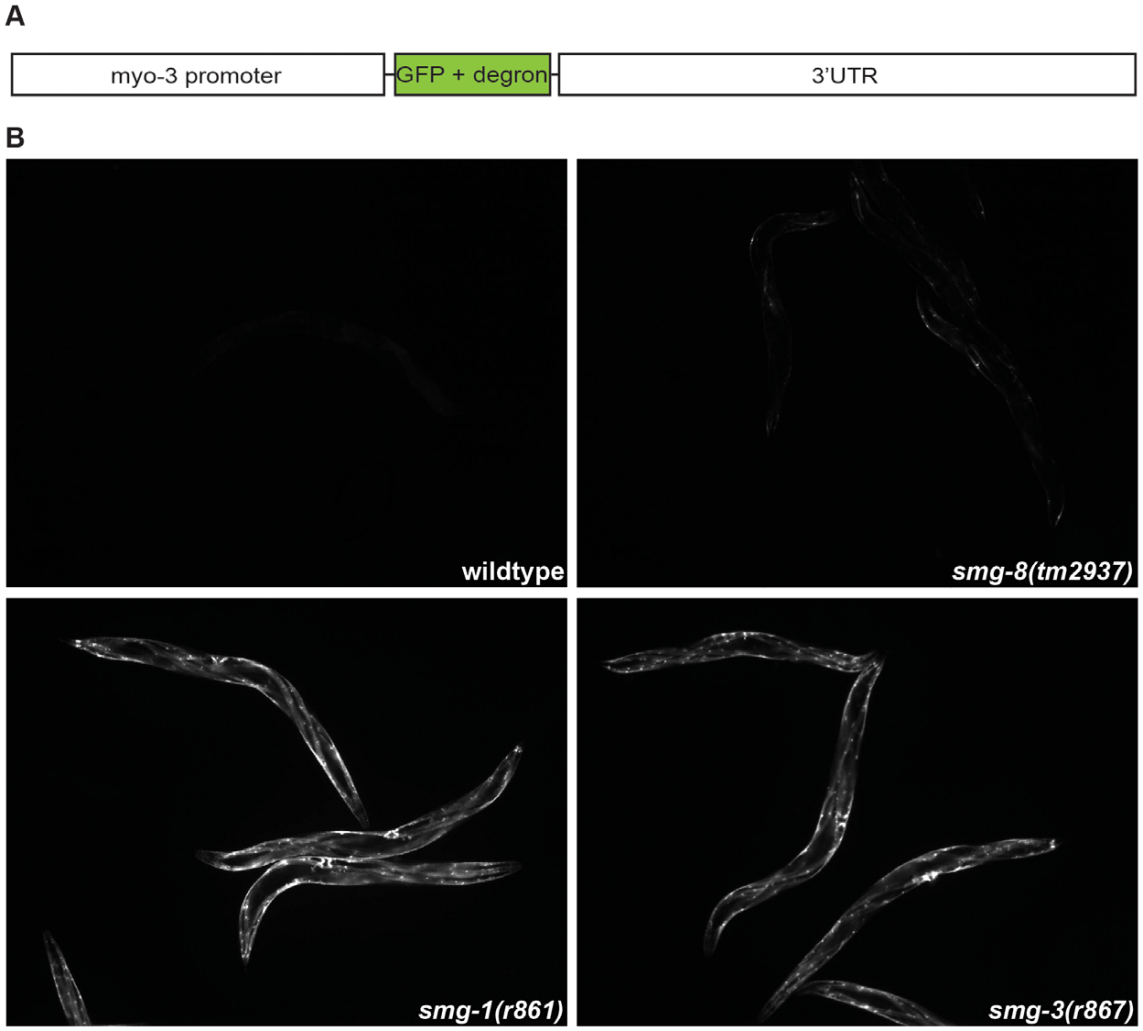
*smg-8* does not restore expression of *myo-3:*:GFP, an exogenous NMD target. (A) Schematic representation of the exogenous NMD GFP reporter, driven by the *myo-3* promoter, which is destabilized by an amino acid sequence that marks a protein for degradation (degron), and a long 3′UTR [24]. (B) *smg-8* and control mutations were introduced into CL724 (*myo-3*::GFP) worms. The double combinations were then inspected under a fluorescent microscope. *smg-1* and *smg-3* mutants express high levels of GFP. In contrast, *smg-8* animals photographed under the same conditions show only a slight accumulation of GFP, similar to the wild type.

As a fourth test for NMD, we examined an exogenous NMD target, *myo-3::GFP*. This strain carries a transgenic GFP reporter that is transcribed in body wall muscles and targeted for degradation by a long 3′UTR (Figure 5A) [24]. When the NMD pathway is inactive, GFP accumulates in muscle fibers, whereas wild-type worms accumulate almost no GFP (Figure 5B). We created double combinations of *myo-3::GFP* and *smg-1*, *smg-3* or *smg-8*. The strain *myo-3::GFP; smg-8(tm2937)* accumulated very little GFP compared to the positive controls *myo-3::GFP; smg-1(r861)* or *myo-3::GFP; smg-3(r867)* (Figure 5B), indicating that *smg-8* is not required for exogenous NMD in *C. elegans*.

In summary, we tested *smg-8* for a role in NMD using four different assays: i) anatomical phenotype and brood size, ii) accumulation of natural NMD targets *rpl-7a* and *rpl-12*, iii) rescue of the paralysis phenotype caused by the endogenous NMD target *unc-54(r293)* and iv) GFP accumulation of the exogenous NMD target reporter *myo-3*::GFP. The discrepancy between our study and the results presented by Yamashita and colleagues is due in part to the use of an allele (our study) vs. RNAi [21]. In addition, we note that the effect of *smg-8* inactivation on NMD in *C. elegans* was not robust in the Yamashita study [21]. In all of our assays, *smg-8* mutants resembled wild-type worms and differed from classical *smg* mutants. We detected no accumulation of mRNA or protein in *smg-8* mutants, even when the *smg-8* mutation was combined with RNAi. We suggest that *smg-8* in *C. elegans* is a novel, conserved gene whose function remains to be elucidated.

## Materials and Methods

### Strains

Worm growth and maintenance were performed as described before [25]. Strains used: SM1618 *unc-54(r293)I*, SM456 *smg-1(r861)I*, SM436 *smg-2(e2008)I,* SM196 *smg-3(r867)IV, smg-1(r861);unc-54(r293)I*, *smg-8(tm2937)II*;*unc-54(r293)I*, CL724 dvIs38 [pCL60 (*Pmyo-3*::GFP::degron/long 3′ UTR)+pRF4], SM1944 *smg-1(r861)I;myo-3*::GFP, SM1929 *smg-3(r867)IV*;*myo-3*::GFP, SM1937 *smg-8(tm2937)II*;*myo-3*::GFP, SM1881 *smg-8(tm2937)II* eight times outcrossed.

### RNA Interference

HT115 bacteria expressing double stranded RNA targeting *smg-1*, *smg-8* or *smg-9* grown for ∼8 hours at 37°C were plated using 1 mM IPTG (Sigma) and 50 mg/ml of Carbenicillin (Sigma). RNAi clones were derived from the Ahringer library [26] and verified by sequencing. Five wild-type, *smg-8(tm2937)* or *eri-6/7(tm1917)* worms were transferred at the L4 stage to RNAi plates and allowed to lay embryos for one day. The progeny was collected ∼48 hours later, when most worms had grown at least to the L4 stage, by rinsing with water and frozen at -80°C for subsequent RNA extraction. Nine 35 mm plates were used per strain per experiment.

### RNA Extraction

For total RNA extraction, glass beads (Sigma) and 1 ml of Trizol Reagent (GibcoBRL) were added to frozen worm pellets. Pellets were lysed by vortex followed by chloroform extraction. RNA was precipitated with isopropanol and washed with 70% ethanol. Resuspended RNA was extracted with phenol:chloroform and precipitated with ethanol. A first-strand reaction kit (NEB) was used to perform the reverse transcriptase reaction, following the manufacturers protocol.

### RT-PCR of *rpl*

Amplification of *rpl-7a* from the cDNA was performed as described in [4], and PCR product was analyzed in a 1% agarose gel. Primers used were rpl-7a-fw GACATCCAGCCAAAGAAGGA and rpl-7a-rv AACGGTGTTTGGTCTCTTGG.

Primers for rpl-12 are Rpl-12-F1 ACCCAAGACTGGAAGGGTCT and Rpl-12 R1 GCCATCGATCTTGGTCTCAT.

### RT-qPCR

For smg-8 and *rpl-7a,* mRNA levels were calculated using the delta-delta-CT method, relative to the control gene *pmp-3* [27]. Control mRNA was normalized to 1 and the mRNA levels are shown as relative fold change. Primers for *smg-8* were smg8-fw-4348 GCTGCCAATATTTCCATCGT and smg8-rv-5165 TGACCACGGGAACATTCATA.

### Brood Size

10 worms at the L4 stage were picked into individual plates at 20°C. Their progeny was counted everyday until no more progeny was generated. The number of progeny per plate was averaged (n = 10).
